# Paired-end small RNA sequencing reveals a possible overestimation in the isomiR sequence repertoire previously reported from conventional single read data analysis

**DOI:** 10.1186/s12859-021-04128-1

**Published:** 2021-04-26

**Authors:** Jose Francisco Sanchez Herrero, Raquel Pluvinet, Antonio Luna de Haro, Lauro Sumoy

**Affiliations:** Institut Germans Trias i Pujol (IGTP), Badalona, Spain

**Keywords:** miRNA, IsomiR, Paired-end sequencing

## Abstract

**Background:**

Next generation sequencing has allowed the discovery of miRNA isoforms, termed isomiRs. Some isomiRs are derived from imprecise processing of pre-miRNA precursors, leading to length variants. Additional variability is introduced by non-templated addition of bases at the ends or editing of internal bases, resulting in base differences relative to the template DNA sequence. We hypothesized that some component of the isomiR variation reported so far could be due to systematic technical noise and not real.

**Results:**

We have developed the XICRA pipeline to analyze small RNA sequencing data at the isomiR level. We exploited its ability to use single or merged reads to compare isomiR results derived from paired-end (PE) reads with those from single reads (SR) to address whether detectable sequence differences relative to canonical miRNAs found in isomiRs are true biological variations or the result of errors in sequencing. We have detected non-negligible systematic differences between SR and PE data which primarily affect putative internally edited isomiRs, and at a much smaller frequency terminal length changing isomiRs. This is relevant for the identification of true isomiRs in small RNA sequencing datasets.

**Conclusions:**

We conclude that potential artifacts derived from sequencing errors and/or data processing could result in an overestimation of abundance and diversity of miRNA isoforms. Efforts in annotating the isomiRnome should take this into account.

**Supplementary Information:**

The online version contains supplementary material available at 10.1186/s12859-021-04128-1.

## Background

MicroRNAs (miRNAs), a class of small non-coding RNAs (ncRNAs), have an average length of 21–23 nucleotides (nt). They have been widely studied as endogenous regulatory molecules that modulate gene expression post-transcriptionally by inducing target mRNA silencing and decay [1]. Additional roles beyond negative modulation of mRNA function have also been proposed [2]. Primary miRNA transcripts (pri-miRNAs) are mainly cleaved by complexes of RNAses III (Drosha and DGCR8) and give rise to one or more precursor miRNAs (pre-miRNAs), also known as hairpins [3]. Following Dicer processing, the hairpins are clipped into short double-stranded RNA. One of the resulting strands (defined as the mature miRNA) binds to the protein Argonaut 2 (Ago2) and gets incorporated into the RNA Induced Silencing Complex (RISC) [4]. Target specificity for binding to mRNAs is mediated by the seed region (defined by miRNA nucleotide positions 2–8) [5], but other parts of the miRNA in central positions and offset bases have also been shown to modulate miRNA functionality [6, 7] (Additional file 1: Fig. S1).

Most miRNA expression studies based on next generation sequencing (NGS) performed to this date have summarized all the reads mapping to a specific miRNA locus or miRNA sequence with or without mismatches and assign it to a single miRNA entity (a miRBase reference database entry). However this type of analysis neglects the fact that not all reads are identical to the mature reference sequence in miRBase [8]. Small RNA sequencing NGS methodology has revealed that miRNAs can frequently appear in the form of multiple sequence variants or isoforms (termed isomiRs) [9, 10]. These isomiRs mainly originate via imprecise or alternative cleavage during pre-miRNA processing and by post-transcriptional modifications [11], including non-templated additions by terminal nucleotidyl transferases [12–14] or editing by adenosine deaminase [15] (Additional file 1: Fig. S1). These changes can influence miRNA stability, sub-cellular localization, target affinity and target specificity [16, 17]. Many reports of isomiRs having more abundant expression than their respective reference canonical miRNA highlight the biological relevance of isomiR variants [18]. Moreover, isomiRs may be more informative than miRNAs as biomarkers for differentiating different cancer types [19] and show differences between genders and ethnicities [20].

Importantly, there is a lack of well established experimental validation methods complementary to NGS with isomiR level resolution. Commercial qPCR assays targeting miRNAs designed to recognize the canonical forms can have variable degrees of specificity for the different corresponding isomiR isoforms [21].

The deviation in miRNA sequences from canonical references has been the focus of many studies correcting technical sources of variation, such as the effect on cross-mapping [22], or minimizing biases and artifacts caused by ligation during library preparation [23]. Comparison between different studies is hampered by the fact that different methods introduce biases in library content due to ligation and subsequent selective amplification of products leading to incomplete or distorted representation of miRNAs profiles [24]. Past studies addressing these issues have been based on SR data analysis. More recently, PE reads analysis was proposed to mine base composition patterns to identify A–I transition edited miRNA relying on error correction through consensus alignment of PE read pairs, but the authors did not look at other types of variation [25]. To address whether base differences found in isomiRs are true biological variations or the result of synthetic artifacts from errors in sequencing, we have compared PE reads with SR reads from small-RNA sequencing experiments.

To the best of our knowledge software tools developed to this date to detect isomiR diversity do not take PE into consideration. This is understandable given that most data is SR mainly due to sequencing cost constraints. We developed XICRA, a pipeline to analyze small RNA sequencing (small RNA-seq) data, which allows detecting and quantifying isomiR level variation in miRNA. As a prerequisite, the pipeline had to be capable of handling both SR and PE reads. It takes compressed fastq files as input, performs a quality check, trims and merges reads in the same pair, maps them using third party tools, such as miraligner [26], sRNAbench [27] or OPTIMIR [28], and generates a gtf file for each sample using miRTOP to annotate isomiRs adopting the latest proposed naming consensus by ‘license plate’ unique identifiers. The pipeline tests for differential expression DE to identify which isomiRs are significantly upregulated or downregulated between conditions. This same functionality was applied to evaluate relative differences between SR and merged PE read counts to assess whether any isomiR sequences could be wrongfully inferred as a result of sequencing errors.

## Results

**Fig. 1:**
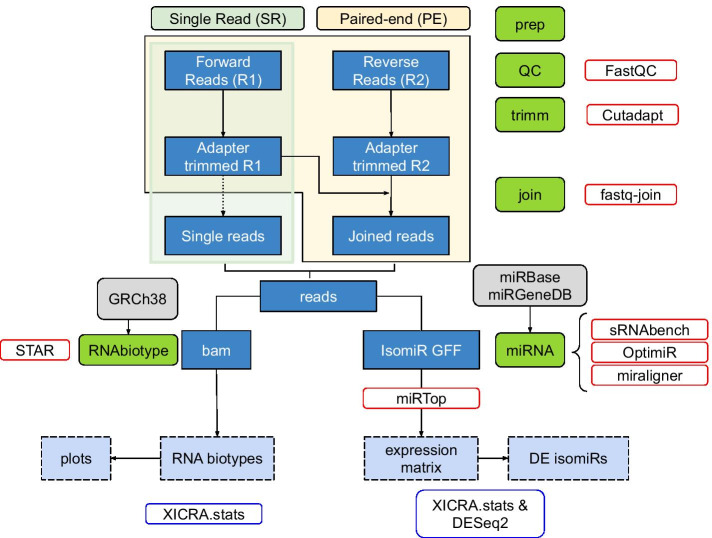
XICRA pipeline workflow: Steps used for the assessment of differential isomiR expression from Single Read (SR) or Paired-end (PE) reads as input. In green are depicted the modules implemented within the pipeline. Dark blue squares represent intermediate results and light blue dashed squares represent final results. Red boxes correspond to the software used in each module and blue boxes correspond to external software to XICRA also used in the analysis of the results

In order to perform systematic analyses of miRNA expression at the isomiR level with error correction functionality based on paired end reads, we built a new pipeline in house named XICRA (see detailed description in the methods section and a scheme in Fig. 1). First, we tested the performance of several tools (including sRNAbench, OPTIMIR and miraligner) on simulated data. Using simulated data allowed us to determine specificity and sensitivity of isomiR detection. One hundred replicates derived from independent runs of simulated read generation were used to generate simulated R1 and R2 reads in silico. For each replicate 100 miRNAs were chosen at random, variant types were randomly generated selecting a single isomiR sequence per variant type. In total 10,000 isomiRs per replicate at defined numbers were used as input into sequencing read simulation software using parameters from the same type of sequencer used in our experimental data (HiSeq2500, Illumina). Comparison of the three tools showed highest sensitivity and specificity using miraligner (Additional files 2, 3: Fig. S2 A, B). We therefore chose to use this software in our subsequent analysis of experimental samples.

We defined SR mode as the isomiR call derived from single reads, and PE mode as the isomiR call derived from the consensus resulting from joining R1 and R2 of the same read pair. When a single read in SR mode maps to an isomiR, while the corresponding merged read in PE mode does not, we can conclude that the isomiR count derived from the SR data analysis on its own is a false positive and should be filtered out.

In an initial exploration comparing simulated reads in SR and PE mode we already detected an overrepresentation of single nucleotide variant (iso_snv) type variants in SR analysis relative to PE analysis. Interestingly R1 data alone appeared to have similar or even enhanced sensitivity to detect isomiR variants but it was less precise, meaning that it led to more false positives than PE sequencing (Additional files 4, 5: Fig. S3 A, B).

Once the pipeline was in place and validated, we went on to test it on real data derived from our experimental samples (Additional file 10: Table S1 shows preprocessing statistics). The first level of analysis looked in more detail for isomiR assignment inconsistencies. Upon inspecting the isomiR type assignment frequencies in SR and PE mode, it was possible to identify deviations in the proportions of some isomiR types. We again observed a higher proportion of filtered reads assigned to isomiRs with an internal SNV (seed SNV or central region SNV isomiRs).

**Fig. 2:**
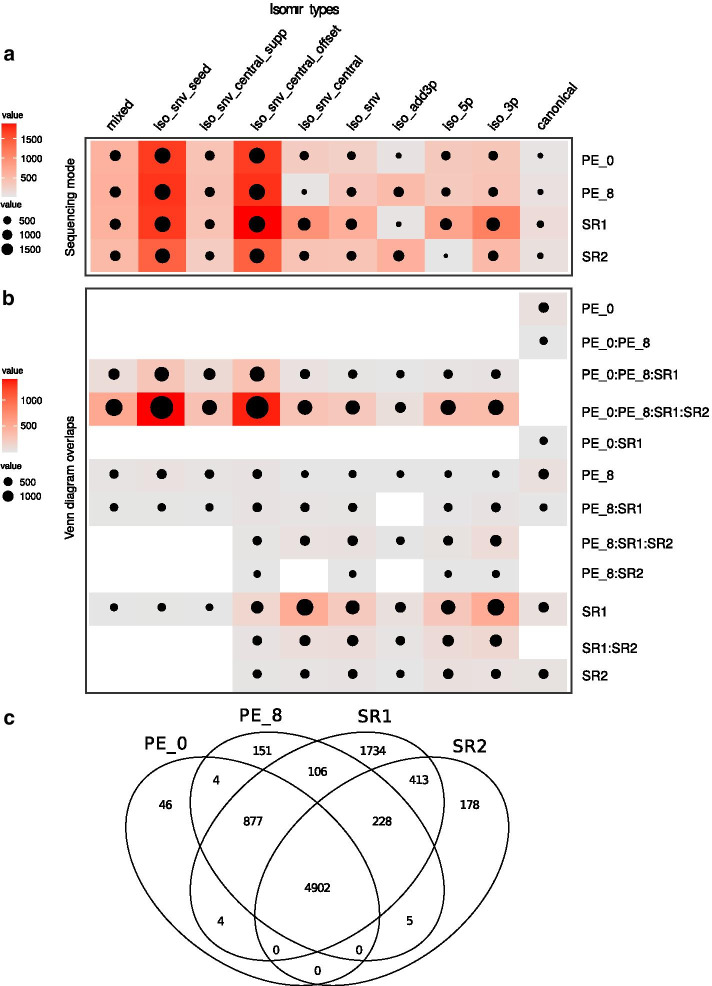
**a** Classification and comparison of unique miRTOP sequences identified for each isomiR class and each category of analysis: PE_0 (PE analysis, parameter fastq-join 0% percentage difference); PE_8 (PE analysis, parameter fastq-join 8% percentage difference); SR1 (single end reads R1) and SR2 (SE reads R2). Mixed isomiR class corresponds to compound miRTOP classes (e.g. snv_seed,iso_add3p; iso_snv_seed,iso3p; etc.) **b** Overlapping results for each isomiR class category showing of unique miRTOP sequences distribution into isomiR types recognized by each sequencing mode. **c** Venn diagram of the overlapping results for all isomiRs classified by method

We further tested the effect of allowing some mismatches (up to 8%, PE_8) or no mismatches (0%, PE_0) in the read pair merging step. Results showed that SR mode calls tend to have an overrepresentation of iso_snv related isomiRs relative to both PE mode calls. We then included two SR mode calls in the comparison (termed SR1 and SR2, from R1 and R2 reads respectively) even though R2 reads on their own would hardly ever be used in real situations. Both SR mode calls yielded a higher proportion of non-canonical isomiR types. Allowing for mismatches led to a higher frequency of these classes possibly due to R2 reads having higher error rates than R1 reads (Fig. 2). IsomiR calls clearly differed between the four different modes (SR1, SR2, PE_0 and PE_8) (Additional file 6: Fig. S4). Also, it was noticeable that most isomiRs that could not be classified by miRTOP univocally due to coexistence of several alterations (“mixed” type) were filtered out. This group of isomiRs was the least represented in the experimental dataset by far. The number of iso_snv variants detected decreased when going from SR1 or SR2 to PE mode under default fastq-join parameters (PE_8). When PE reads were merged without allowing any errors (PE_0) the total number of reads assigned to iso_snv classes further decreased. This difference between the result with PE_8 and PE_0 would suggest that tolerance to mismatches between read pairs should be avoided when analyzing isomiR diversity based on PE data.

**Fig. 3:**
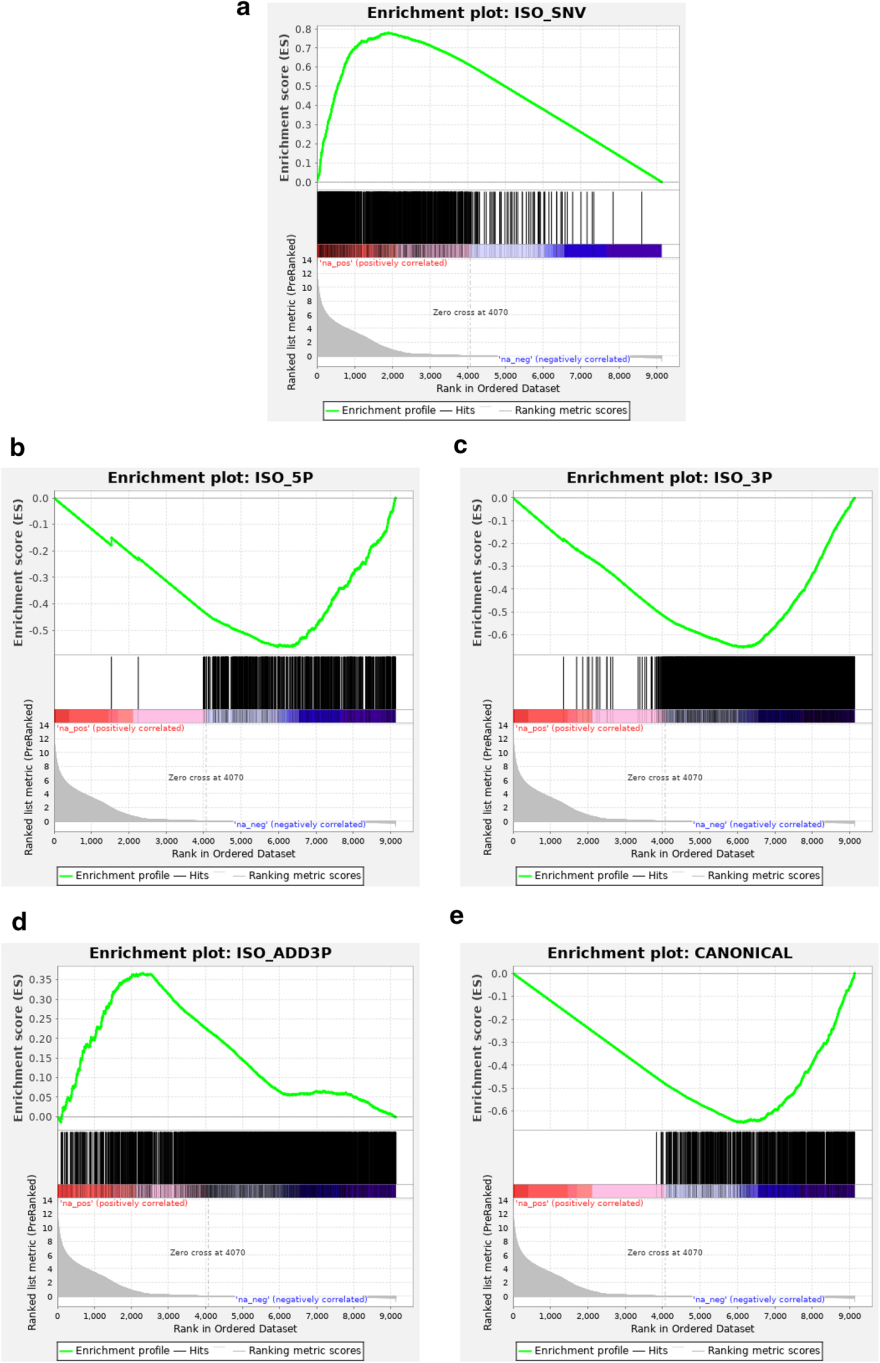
GSEA enrichment plots. Pre-ranked GSEA analysis was performed using as metric the normalized log2FoldChange estimate from DESEq2 between SR and PE datasets from the same samples (30 normal serum samples, filtering for miRNA and isomiR sequences present in at least 10 samples). Enrichment of each class of isomiR was assessed. Only iso_snv class isomiRs showed significant enrichment. **a** iso_snv, **b** iso_5p, **c** iso_3p, **d** iso_add3 **e** canonical enrichment plots. Please see Table 1 for complete output results

We went beyond a descriptive analysis by applying differential miRNA expression analysis methodology. Results showed many iso_snv-related variant classes to be differentially expressed in SR versus PE mode, both by DESeq and by GSEA (Fig. 3, Table 1). However, the impact was almost negligible in terms of the fraction of total reads affected, as the expression level of these differentially expressed iso_snv isomiRs were very low (Additional file 7: Fig. S5).

**Table 1:** GSEA output results for isomiR type enrichment in SR versus PE data comparison. Size corresponds to the number of unique sequences detected from each isomiR type; enrichment Score (ES) and False Discovery Rate q-value (FDR q-value) are as provided by GSEA analysis

Name	Size	ES	FDR q-val
iso_snv_seed	1116	0.788	0.073
iso_snv	946	0.779	0.040
iso_snv_central	664	0.748	0.034
iso_snv_central_supp	799	0.736	0.028
iso_snv_central_offset	151	0.725	0.025
mixed	1	0.567	1.000
iso_add3p	2232	0.367	1.000
iso_5p	552	− 0.563	1.000
canonical	691	− 0.649	1.000
iso_3p	1986	− 0.654	1.000

Searching the literature and genomic databases we were only able to identify a few public miRNA sequencing datasets with PE data. We tested if our observation could be made in data from a published study on non-alcoholic fatty liver disease [29] (Additional file 11: Table S2 shows preprocessing statistics). The results (Additional files 8, 9: Fig. S6 A, B) show similar trends and behavior.

## Discussion

Because of cost considerations most studies in the literature looking at miRNA by NGS use SR only. We hypothesized that analysis of small RNA-seq PE data at the isomiR level is likely to contribute to discriminating resolution improvements in miRNA differential expression analysis. We had small RNA libraries sequenced in PE mode derived from healthy human serum samples. This offered us the opportunity to evaluate how much the results of an isomiR analysis could improve with PE reads compared to an analysis which only used the forward SR1 (or the reverse SR2). Our results would support the notion that PE sequencing may lead to more exact profiling at the cost of not detecting a few variants that are detected by SR sequencing alone which may be in regions difficult to sequence, which often have even poorer sequence quality from the alternate strand.

We tested two different conditions for PE read pair merging. The default of fastq-join is to allow up to 8% errors in the alignment between two paired reads (which for miRNAs would entail 1 or 2 nucleotide differences in ~ 21 bp). This could in principle lead to retention of sequencing errors in cases of ambiguity and low quality in both reads or with a call in R2 missing altogether (resulting in a conflicting position being incorrectly resolved in favor of the mismatch to the miRBase mature miRNA reference).

Comparing SR1 versus SR2 performance showed that R2 accumulates many more errors and SR2 based leads to higher inferred isomiR diversity than SR1 mode analysis. Differences in quality along the length of the reads may explain why some particular isomiR type frequencies differed depending on the reads used. Illumina quality tends to dip the first few cycles due to the nature of the base caller calibration procedure used in Illumina sequencing by synthesis. Based on our results it is possible to hypothesize that R1 may be enriched in artifactual iso_snv isomiRs with SNVs in the seed region due to the slightly higher error rate in the first few bases. R2 on the contrary may tend to have errors in the 3′ end of the miRNA and isomiR sequences. Cancelling out these errors through PE_8 mode, with mismatch allowance, leads both to correcting iso_snv variants derived from R1 read errors, and to enhancing iso_3add and iso_3p variants (derived from R2 read errors). PE-0 mode, with zero mismatch tolerance in the joining step, would be the most conservative and closer to the truth and would be recommended at the cost of lower count yields.

The mechanism triggering this may be that R1 reads mapping to iso_snv isomiRs may be intrinsically of poorer quality and may not have R2 read pairs of sufficient quality and are not eligible for fastq joining step. Alternatively and less likely R2 in the same read pair matching the canonical miRNA sequence should be of better quality than R1. This would support the hypothesis that inflated artifactual iso_snv counts are derived from sequencing errors in poor quality reads.

GSEA analysis of isomiR type enrichment when comparing SR versus PE would suggest that the enrichment in iso_snv appears concomitant to depletion in canonical isoforms (implying that when using the iso_snv isomiRs which would be called by SR are assigned to canonical when in PE mode).

Although in our particular results iso_snv isomiRs represented a fairly small proportion of differentially expressed isomiRs between male and female samples, in other studies it may still be worthwhile to test this in order to take into account those isomiRs that are artifactual, provided that samples have been sequenced in PE mode. Alternatives such as the use of unique molecular identifiers (UMIs) have been proposed to eliminate PCR duplicates and correct for sequencing errors but the cost involved by the inclusion of special adapters and additional required sequencing depth may justify using PE reads instead.

Our work does not allow inferring conclusions on the impact of SR data prevalence in results in the literature on mature miRNA profiling and isomiRs reported as differentially expressed. It is possible to hypothesize that data derived from PE sequencing could help reduce noise and artifacts and prevent any false positive results that may have derived from SR data.

Because the seed and central region of the isomiRs is the region that interacts with the mRNA, the finding that internal SNV isomiRs calls are most likely generated by sequencing errors could impact subsequent analyses and bias the derived conclusions regarding putative functional consequences. The results of our study are relevant because of the importance given to isomiRs with SNVs in the seed region for miRNA regulatory functions at the biological and mechanistic level. In addition, our findings could help to address the challenges that isomiR variation imposes on the development of validation and diagnostic assays for miRNA biomarker assessment by helping to define true isomiRs.

Our study has limitations in that it only addresses noise due to sequencing errors revealed by mismatched read pairs, and is subject to biases in the software tools used. Besides and bioinformatic analysis data processing, additional sources of variation affecting isomiR profiling may come from key factors in library preparation such as enzymatic steps, adapter sequences and size selection procedures. Optimization of these different parameters is dependent on the use of controls and validation by alternative methodologies. Current state of the art makes isomiR data results inherently noisy and hard to verify. Widespread adoption of data formats and nomenclature such as miRTOP implemented in our pipeline XICRA will be required in order to be able to compare different studies. Reanalysis of older datasets may make it possible to recover and reinterpret previous work. Convergence to optimal analysis strategies and software standardization will be needed. Future work in the field should address all these issues. Concerted efforts should help to improve our understanding of miRNA biology and aid in identifying miRNA based biomarkers by exploiting and mining true isomiR diversity.

## Conclusions

We can conclude that PE sequencing improves isomiR calling in small RNA sequencing data.

Internal variation isomiR calls are frequent artifacts in SR sequencing data. Gene set enrichment analysis by GSEA of isomiR classes pre-ranked by SR vs. PE log2 fold change shows significant iso_snv enrichment in SR data compared to PE data. Systematic differences between SR and PE mode sequencing affecting putative internally edited isomiR (iso_snv) levels exist. Many detectable internal sequence variant isomiRs in SR mode may be false positives.

A significant fraction of differentially expressed miRNAs in serum in SR data are iso_snv isomiRs and can be filtered out using PE mode. Many of these are found at very low expression levels and often are removed by thresholding or universal filtering steps in DESeq2 DE analysis. In contrast, most differentially regulated canonical miRNA and length variant isomiR types (iso_5p, iso_3p and iso_add3) can be reliably detected in SR data. Many more isomiRs than canonical miRNAs are found regulated.

Internal RNA editing miRNA results should be taken with caution. Most putative internal sequence variants detected in SR mode sequencing could be artifactual. This implies RNA editing may not be as prevalent in isomiR biogenesis as proposed. SR sequencing, with iso_snv filtering, may be sufficient for isomiR resolution. PE sequencing produces better quality reads. However, PE’s higher cost than SR precludes use in most studies. Filtering out iso_snv hits from SR data may be indicated for isomiR level analysis for most studies.

Validation of isomiRs by alternative methods would be advisable especially for iso_snvs derived from SR datasets, but this may be technically unfeasible or inefficient because of the high false positive rate. Continually decreasing sequencing costs may allow adoption of PE sequencing as the preferred method in future miRNA studies focusing on isomiR variation.

## Methods

### RNA extraction, library preparation and Illumina sequencing

We extracted total RNA from 30 serum samples from healthy individuals, anonymous blood donors who consented to collection of a separate fraction for circulating RNA biomarker discoveries with the miRCURY RNA Isolation kit for Biofluids (Exiqon) spiked in with Exiqon RNA spike in mix (1:50) of recommended amount eluted in 50 µl. As RNA concentration was too low to be measured by spectrophotometry or fluorometry, quality of RNA was assessed with the microRNA QC PCR Panel, 96 well (V1.RO) from 5 µl of 1:50 dilution of cDNA obtained after reverse transcribing 2 µl of total RNA with miRCURY LNA™ Universal RT microRNA PCR (Exiqon) which uses external spiked in to control for miRNA yield and profiles specific miRNAs to estimate hemolysis. Quantitative PCR reactions were performed on Roche LightCycler 480 instrument (Roche) in 10 µl volume reactions. All samples included in the analysis had equivalent miRNA content and low levels of hemolysis (ΔCq (miR-23a–miR-451) ranging from 4.46 to 6.47, below the assay rejection limit value of 7). We constructed indexed libraries using the TruSeq Small RNA kit (Illumina) starting from all remaining RNA extracted from 200 µl (~ 48 µl, equivalent to ~ 192 µl serum). Library products were pooled together in equimolar amounts based on Bioanalyzer peak quantification at the expected size around 142 bp. A single pool with 30 libraries was size selected with the Pippin prep system (SAGE Science) with 3% agarose and dye free Marker F in the 115–165 bp broad range to enrich for miRNA products and minimize variation between samples in the size selection step. The sequencing data generated consisted of 2 × 50 nt reads resolved on a HiSeq 2500 system with v3 SBS reagents (Illumina). Two of the 30 samples were excluded because they were clear outliers by PCA exploratory analysis and had poor library yields. The final dataset consists of 28 samples.

### Small RNA-seq analysis pipeline

To analyze small RNA-seq PE data we have developed the XICRA pipeline (https://github.com/HCGB-IGTP/XICRA). It is developed in python, with multiple separate modules and it is available as a pip package (https://pypi.org/project/XICRA/). This pipeline is designed to take paired end reads in fastq format, trim adapters and low-quality base pairs positions, and merge read pairs (R1 & R2) that overlap. A mapping step to the reference genome (user defined) assigns joined reads to all major RNA biotypes including miRNA and isomiRs, tRNA fragments (tRFs) and piwi associated RNAs (piRNAs). Then, XICRA produces a miRNA analysis at the isomiR level using joined reads, with several choices of software that can be selected by the user with standardized output. Results are generated for each sample, analyzed and summarized for all samples in a single expression matrix. This information can be processed at the miRNA or isomiR level (single sequence) but also summarizing for each isomiR variant type. Statistical summaries can be easily accessed using the accompanied R package XICRA.stats (https://github.com/HCGB-IGTP/XICRA.stats). Although the pipeline is designed to take paired-end reads, it also accepts single-end reads. The workflow of the pipeline is described in Fig. 1.

XICRA uses cutadapt [30] for the adapter trimming analysis. Default trimming preset parameter settings are: to keep all reads regardless of whether the adapter is found or not, a 10% maximum adapter matching error rate (mismatches, insertions and deletions), and a 3 bp minimum overlap length. User must provide specific adapter sequences for the trimming analysis. An optional previous quality checking step can be performed for each sample using FastQC [31] before the trimming analysis. Results are summarized for all samples using MultiQC software [32].

Once all reads are adapter trimmed, the tool uses fastq-join from ea-utils [33] to join the two PE reads, if provided, on the overlapping ends. Apart from the joined reads, this tool also generates two files with the R1 and R2 reads that cannot be joined. As a default the minimum overlap is set to 6 bp and the maximum allowed difference for the reads to be joined is set to 0% to retain 100% matching read pairs ensuring high quality sequencing information. Parameters can be modified using the different options provided.

The XICRA pipeline can continue to process either joined PE reads or SR reads. Two levels of mapping are implemented. The first level profiles RNA biotypes using STAR [34] to map reads against the reference genome and featureCounts [35] to extract and quantify numbers of reads by class. The second level focuses specifically on small RNA subclasses. Here we describe the miRNA analysis implemented within XICRA but the modularity and versatility of the pipeline would make it quite straightforward to include other RNA biotypes analyses in detail.

For miRNAs analysis at the isomiR resolution level, XICRA allows the user to use either miraligner [26], sRNAbench from sRNAtoolbox [27] or OPTIMIR [28]. Each software uses different strategies and might produce different results [36]. We have included them as they allow following standardization procedures performed by miRTOP software and adopt the miR.gff3 file format [37]. Again, the pipeline modular implementation would allow adding additional softwares converging and adapting to miRTOP and miR.gff3 format. For each of the softwares mentioned above and included within the miRNA module in XICRA default parameters are used. Some of these parameters can be modified using the different options provided. As a result of this miRNA module, annotation is generated that categorizes isomiRs into classes based on their sequence modifications (including iso_5p, iso_3p, iso_add, iso_snv, iso_snv_seed, iso_snv_central_offset, iso_snv_central, iso_snv_central_suppl) following miRTOP suggested classification scheme. A final conversion step from individual per sample miR.gff3 files into a single expression matrix is performed. This file serves as input for differential expression (DE) analysis. Information is provided for each unique sequence and indexed names contain the miRNA, the variant type and license plate (unique identifier, UID) provided by miRTOP. Duplicated entries at the sequence level, produced by different modifications from the same or different miRNA are discarded. An additional matrix is provided containing the sequence information for each encrypted UID.

Per sample read count matrices at the isomiR level are summarized into a single expression matrix that it serves as input for DE analysis between the comparison groups of interest. We have generated an additional R package (XICRA.stats) that facilitates the retrieval of these matrices and parses the information included within each unique index name provided. The DE analysis can be done aggregating data at the mature miRNA level (i.e. hsa-miR-501-3p), by isomiR class (i.e. hsa-miR-501-3p_iso_5p), by specific length variant cluster (i.e. hsa-miR-501-3p_iso_3p:-2) or with the sequence of the read itself as the counting data. This is useful since different types of modification may coexist in a single sequence, and non-templated additions and internally edited sequences can differ leading to isomiRs that can fall into different categories or be derived from different mature miRNAs. DE analysis is performed outside of the tool with DESeq2 package in R [38].

### Gene set enrichment analysis applied to isomiRs

We adapted the Gene Set Enrichment Analysis (GSEA) tool [39] for miRNA analysis by building miRNA isoform ‘gene sets’ grouping all class identifiers by class type (including iso_5p, iso_3p, iso_add, iso_snv, iso_snv_seed, iso_snv_central_offset, iso_snv_central, iso_snv_central_supp). Gene expression results were issued for pre-ranked mode analysis with the default setting sorted by the log2fold change metric. Gene sets were built from the isomiRnome detected in serum from male and female individuals, the dataset generated in house used in this study. All unique sequences obtained after trimming all reads in all samples combined were annotated at the isomiR level and grouped by isomiR type. Each isomiR gene set was composed of all the unique sequences assigned to a particular isomiR type.

### Venn diagrams

Venn diagrams depicting overlap between regulated genes were made using the Venn function in the gtools package in R [40].

### External paired end read small RNA sequencing dataset

We searched the NCBI SRA database for any small RNA sequencing datasets generated using Illumina paired end reads. Very few datasets were found fulfilling our filtering criteria. We found a dataset belonging to a project (GEO GSE114923; PRJNA473134) that studied miRNA biomarkers in nonalcoholic fatty acid liver disease (NAFLD). The design of the experiment contained 8 patients, classified as control or case, and the dataset was sequenced using paired-end NextSeq 500 paired-end libraries. See additional details in the original publication [29].

We produced a full analysis at the isomiR level using XICRA and the three different software available (sRNAbench, miraligner and OPTIMIR) using paired-end information and single end reads (R1 and R2, respectively). For the trimming process, we used as adapter sequences the corresponding NEBNext library adapters. Once the isomiR expression matrices were generated for each software and read type, we used R package XICRA.stats for parsing the information and DESeq2 in R [38] for the analysis of DE isomiRs or miRNAs using the experiment design described above (case vs. control).

### Computer simulations

To illustrate the potential of paired-end reads at the isomiR level analysis we have generated computer simulations to test the impact of technical errors from single end or paired-end reads. We followed the guidelines previously described for isomiR computer simulations by Amsel et al. 2017 [36]. We created biological variation and technical variation using multiple high throughput sequencing profiles and evaluated the performance of the simulation using sensitivity and precision of the isomiRs detected under several circumstances. See details of the bioinformatic script and details in (https://github.com/HCGB-IGTP/XICRA/tree/master/BMC_bioinformatics_paper/simulation).

#### Biological variation

We created artificial miRNA isoforms from *Homo sapiens* mature and hairpin sequences in miRBase (v.21) [41]. We additionally included the canonical fasta sequence of each miRNA to the variant dataset generated. From the variant frequency table generated we selected 100 miRNAs and discarded some random variants and generated random distribution frequencies of the variant types generated. To simplify the interpretation and evaluation, for each variant type, we selected a single isoform. For each variant type we included the corresponding frequencies generated to a total amount of 100 sequences for each mature miRNA. Thus a total of 10,000 sequences were simulated with preset frequencies.

#### Technical simulation

For each biological dataset generated, we used ART [42] with Illumina HiSeq2500 and MiSeq-v1 sequencing system profiles using paired-end mode to simulate next generation sequencing (NGS) reads. We grouped all isoforms according to length and generated NGS simulation for each length subset to finally merge them all in a single file for each read accordingly. We used a 10 × sequencing coverage for each input fasta sequence. As previously noted [36], due to the nature of the ART simulation we had to parse and omit about half the total reads generated as they were reverse complemented. We only discarded reverse complemented R1 reads and its R2 counterpart accordingly. Due to the implementation based on frequencies that we did in the biological variation procedure, we made sure that, when applying the same coverage for each sequence and discarding reverse complement, the biological variation frequencies generated would be maintained in the NGS simulation. The observed range would vary from 5–500 counts for each single variant type simulated.

#### Performance evaluation

We evaluated the performance of using PE reads for miRNA isomiR analysis using the NGS simulation datasets and the pipeline XICRA. For each dataset, we used the miRNA module using paired-end mode and single end mode for the R1 and R2 reads. For the paired-end mode we initially joined reads using two different join percentage difference cutoff (fastq-join parameter) to test the effect of using 100% perfect R1 and R2 reads or allowing the default difference (8%) along the minimum default overlap length cutoff (6 bp). For the single-end mode, we used the total R1 reads simulated or the total R2, reversed complemented using seqtk software [43] respectively. We generated a miRNA analysis at the isomiR level using the three different softwares available within XICRA: miraligner, sRNAbench and OPTIMIR.

Using the biological variation frequencies generated as true positives for each dataset, we evaluated the amount of isomiRs detected for each software and type of read. For paired-end reads, we also used a different percentage difference cutoff. For each isomiR, the detected counts were classified as: True positives (TP) when observed counts matched the expected counts; false positives (FP) when observed counts exceeded the expected counts and were wrongly assigned; and false negatives (FN) when observed counts did not get to the minimum expected counts. We calculated the sensitivity or recall as TP/(TP + FN) and the precision or specificity as TP/(TP + FP). We also reported True Negatives (TN) when expected counts were not observed and new generation isomiRs when new variants or miRNA appeared and were not expected. We plotted results using ggplot2 [44] for each software and type of read respectively.

## Supplementary Information

**Additional file 1:** Figure S1: Schematic summarizing the biogenesis of miRNA and isomiRs (Adapted from figure 1 in [45] with permission of the Association for Research in Vision and Ophthalmology, the copyright holder, and freely accessible Wikipedia image content [46]).

**Additional file 2:** Figure S2 A: Simulation statistics for three different softwares, from left to right (miraligner, OPTIMIR and sRNAbench) and in colors for different read types: red, Paired-end (PE); green, Single-end R1 (R1) and blue, SE R2 (R2). Statistics are from top to bottom, Sensitivity, calculated as TP/(TP+FN); Precision or specificity, calculated as TP/(TP+FP); and New Generation as the total count of new non-expected isomiRs detected. We show results using PE reads joined with 0% fastq.

**Additional file 3:** Figure S2 B: Simulation statistics for three different softwares, from left to right (miraligner, OPTIMIR and sRNAbench) and in colors for different read types: red, Paired-end (PE); green, Single-end R1 (R1) and blue, SE R2 (R2). Statistics are from top to bottom, Sensitivity, calculated as TP/(TP+FN); Precision or specificity, calculated as TP/(TP+FP); and New Generation as the total count of new non-expected isomiRs detected. We show results using PE reads joined with 0% fastq.

**Additional file 4:** Figure S3 A: Classification and comparison of miRTOP sequences from simulations results using miraligner software, for each isomiR class and each category of analysis: PE_0 (PE analysis, parameter fastq-join 0% percentage difference); PE_8 (PE analysis, parameter fastq-join 8% percentage difference); SR1 (single end reads R1) and SR2 (SE reads R2). We represented the total average read counts (A) and the count of unique isomiRs detected (B).

**Additional file 5:** Figure S3 B: Classification and comparison of miRTOP sequences from simulations results using miraligner software, for each isomiR class and each category of analysis: PE_0 (PE analysis, parameter fastq-join 0% percentage difference); PE_8 (PE analysis, parameter fastq-join 8% percentage difference); SR1 (single end reads R1) and SR2 (SE reads R2). We represented the total average read counts (A) and the count of unique isomiRs detected (B).

**Additional file 6:** Figure S4: Venn diagrams of the overlapping isomiRs identified for each category of analysis and subclassified for each isomiR type category: A) canonical; B) iso_3p; C) iso_5p; D) iso_add3p; E) iso_snv; F) iso_snv_central; G) iso_snv_central_offset; H) iso_snv_central_supp; I) iso_snv_central_seed; J) mixed category.

**Additional file 7:** Figure S5: Overlapping results for each isomiR class category showing of overall read cumulative read counts distribution into isomiR types recognized by each sequencing mode.

**Additional file 8:** Figure S6 A: Classification and comparison of miRTOP sequences from GEO project GSE114923 using miraligner software, for each isomiR class and each category of analysis: PE_0 (PE analysis, parameter fastq-join 0% percentage difference); PE_8 (PE analysis, parameter fastq-join 8% percentage difference); SR1 (single end reads R1) and SR2 (SE reads R2). We represented the total average read counts (A) and the count of unique isomiRs detected (B).

**Additional file 9:** Figure S6 B: Classification and comparison of miRTOP sequences from GEO project GSE114923 using miraligner software, for each isomiR class and each category of analysis: PE_0 (PE analysis, parameter fastq-join 0% percentage difference); PE_8 (PE analysis, parameter fastq-join 8% percentage difference); SR1 (single end reads R1) and SR2 (SE reads R2). We represented the total average read counts (A) and the count of unique isomiRs detected (B).

**Additional file 10:** Table S1: Small RNA sequencing data processing statistics for all 28 serum samples from blood donor sequenced in house (GSE155370; ).

**Additional file 11:** Table S2: Small RNA sequencing data processing statistics for all 16 plasma samples from NAFLD patients from a public dataset (GSE114923).

## Data Availability

Data generated in this work can be accessed at https://www.ncbi.nlm.nih.gov/geo/query/acc.cgi?acc=GSE155370. Analysis pipeline is accessible https://github.com/HCGB-IGTP/XICRA. Simulated data and scripts are located in https://github.com/HCGB-IGTP/XICRA/tree/master/BMC_bioinformatics_paper. Additional external dataset summary annotation and procedural information is available at: https://www.ncbi.nlm.nih.gov/geo/query/acc.cgi?acc=GSE114923, and raw sequencing data was downloaded from https://www.ncbi.nlm.nih.gov/bioproject/?term=PRJNA473134. Reads analyzed in this study were mapped to the human genome reference GRCh38.87 (ftp://ftp.ensembl.org/pub/release-87/gtf/homo_sapiens/Homo_sapiens.GRCh38.87.gtf.gz) and to the miRBase v21 hairpin and mature microRNA reference databases (ftp://mirbase.org/pub/mirbase/21/).

## Abbreviations

DE: Differential expression
GSEA: Gene set enrichment analysis
NGS: Next generation sequencing
PE: Paired-end
PE_0: Paired-end mode with 0 mismatches allowed prior to merging
PE_8: Paired-end mode with 8% mismatches allowed prior to merging
SR: Single read
SR1: Single read mode from R1 reads
SR2: Single read mode from R2 reads
R1: Read 1
R2: Read 2
SNV: Single nucleotide variant
