# Supplementary figures and images for: Paired-end small RNA sequencing reveals a possible overestimation in the isomiR sequence repertoire previously reported from conventional single read data analysis
Source: BMC Bioinformatics. 2021 Apr 26;22:215. doi: 10.1186/s12859-021-04128-1 (PMC8077951; doi:10.1186/s12859-021-04128-1)

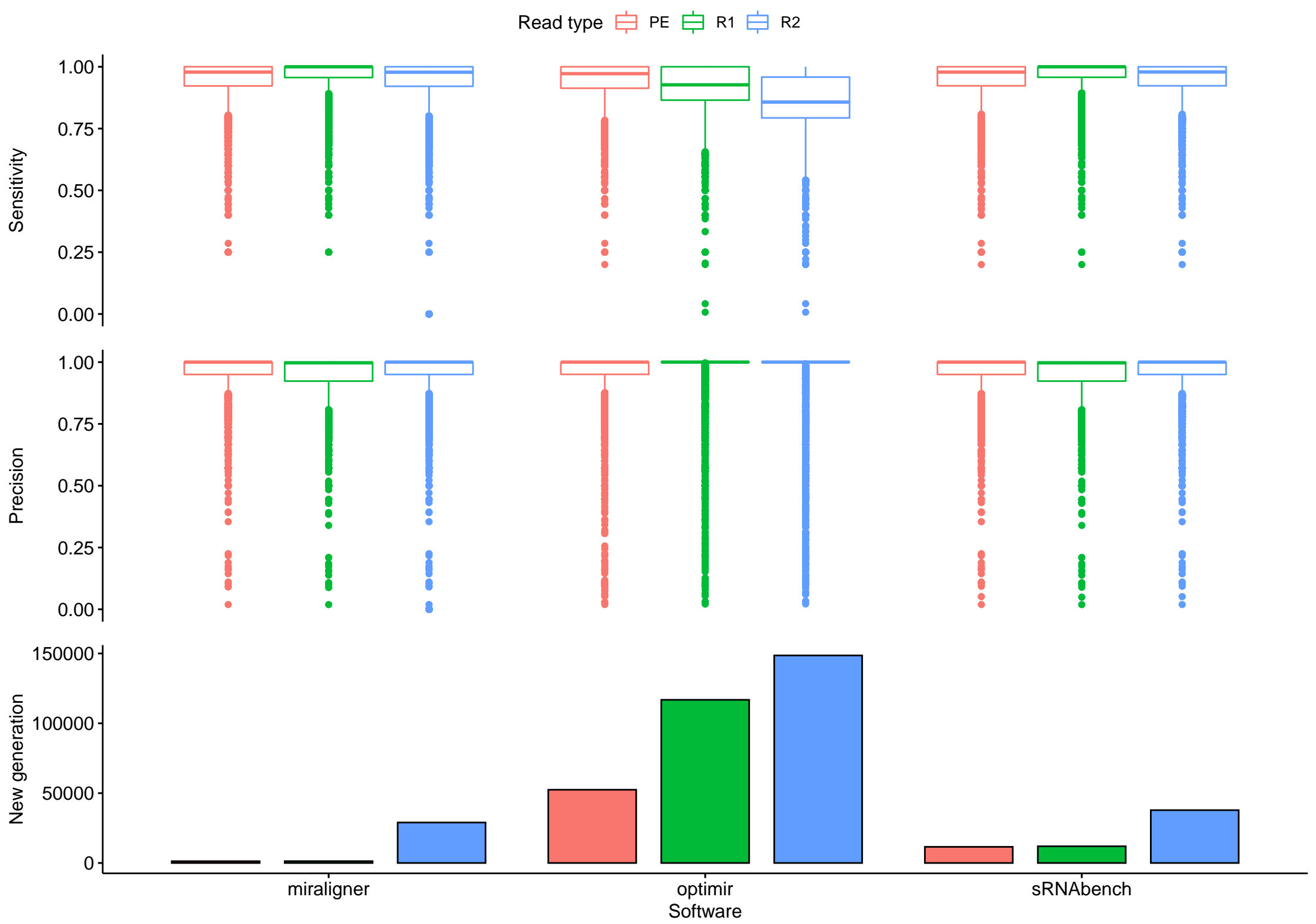

Supplement: Supplementary file 2 — Additional file 2: Figure S2 A: Simulation statistics for three different softwares, from left to right (miraligner, OPTIMIR and sRNAbench) and in colors for different read types: red, Paired-end (PE); green, Single-end R1 (R1) and blue, SE R2 (R2). Statistics are from top to bottom, Sensitivity, calculated as TP/(TP+FN); Precision or specificity, calculated as TP/(TP+FP); and New Generation as the total count of new non-expected isomiRs detected. We show results using PE reads joined with 0% fastq. [file 12859_2021_4128_MOESM2_ESM.pdf]

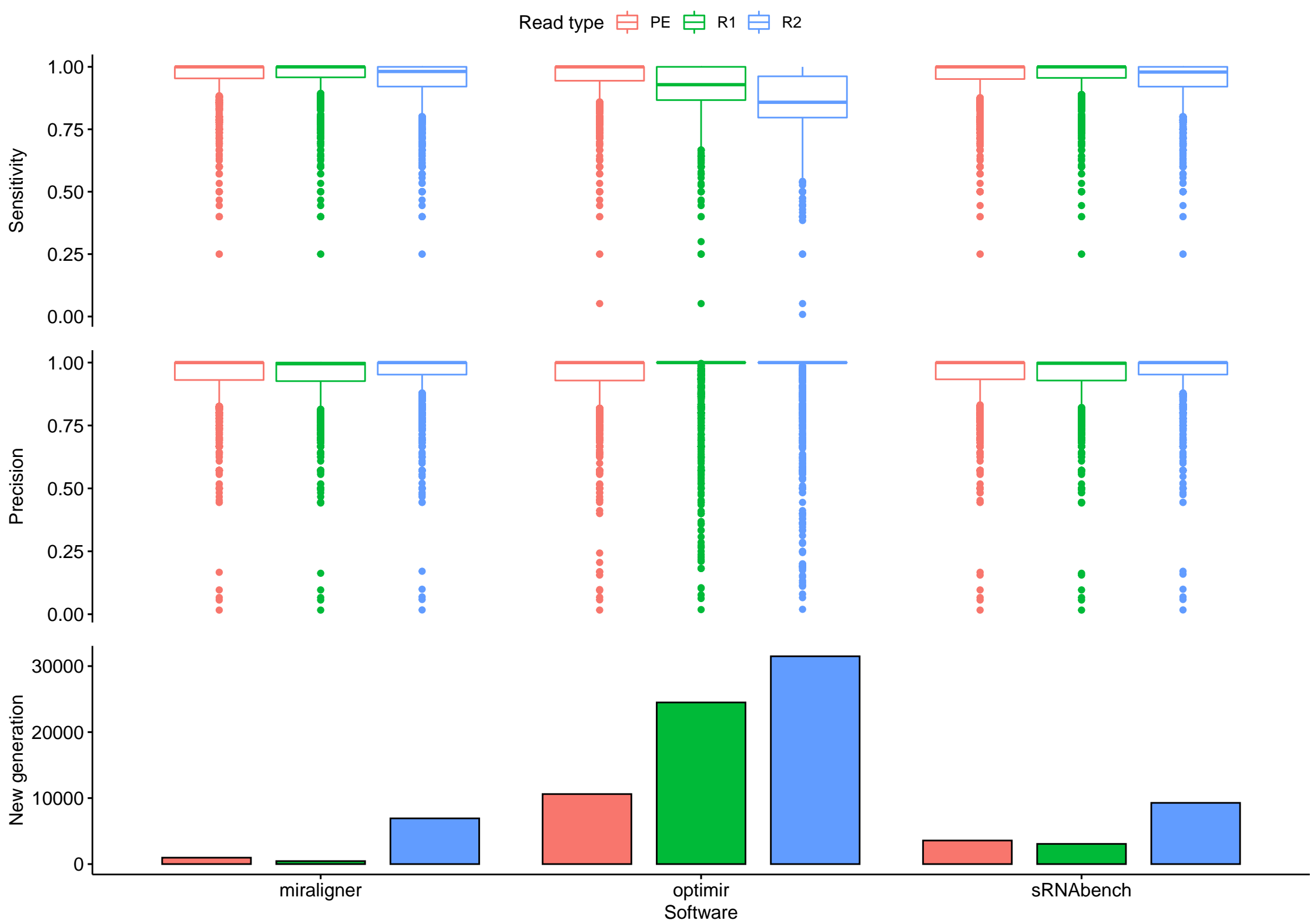

Supplement: Supplementary file 3 — Additional file 3: Figure S2 B: Simulation statistics for three different softwares, from left to right (miraligner, OPTIMIR and sRNAbench) and in colors for different read types: red, Paired-end (PE); green, Single-end R1 (R1) and blue, SE R2 (R2). Statistics are from top to bottom, Sensitivity, calculated as TP/(TP+FN); Precision or specificity, calculated as TP/(TP+FP); and New Generation as the total count of new non-expected isomiRs detected. We show results using PE reads joined with 0% fastq. [file 12859_2021_4128_MOESM3_ESM.pdf]

## Average counts per isomir type

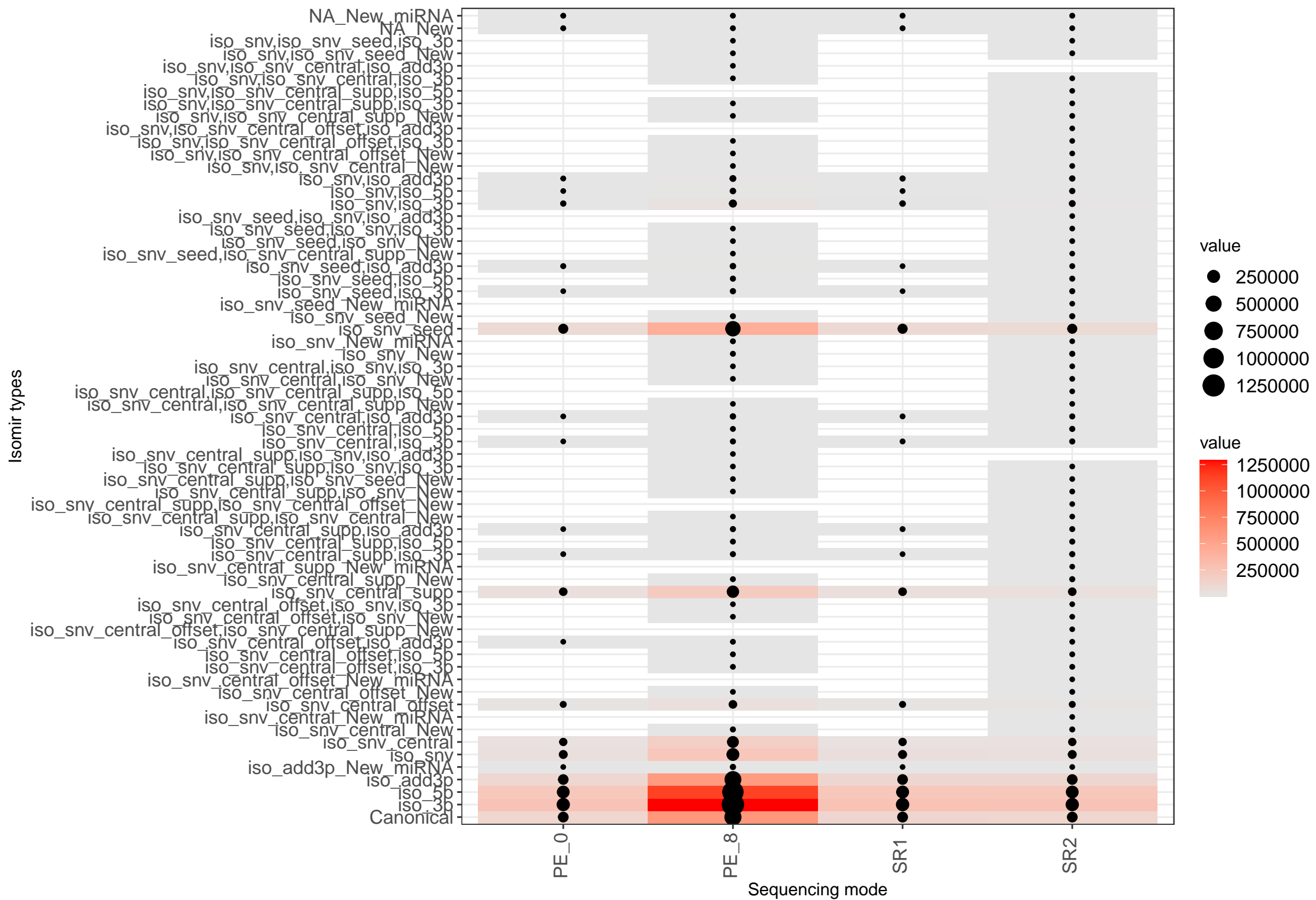

Supplement: Supplementary file 4 — Additional file 4: Figure S3 A: Classification and comparison of miRTOP sequences from simulations results using miraligner software, for each isomiR class and each category of analysis: PE_0 (PE analysis, parameter fastq-join 0% percentage difference); PE_8 (PE analysis, parameter fastq-join 8% percentage difference); SR1 (single end reads R1) and SR2 (SE reads R2). We represented the total average read counts (A) and the count of unique isomiRs detected (B). [file 12859_2021_4128_MOESM4_ESM.pdf]

## Unique isomiRs

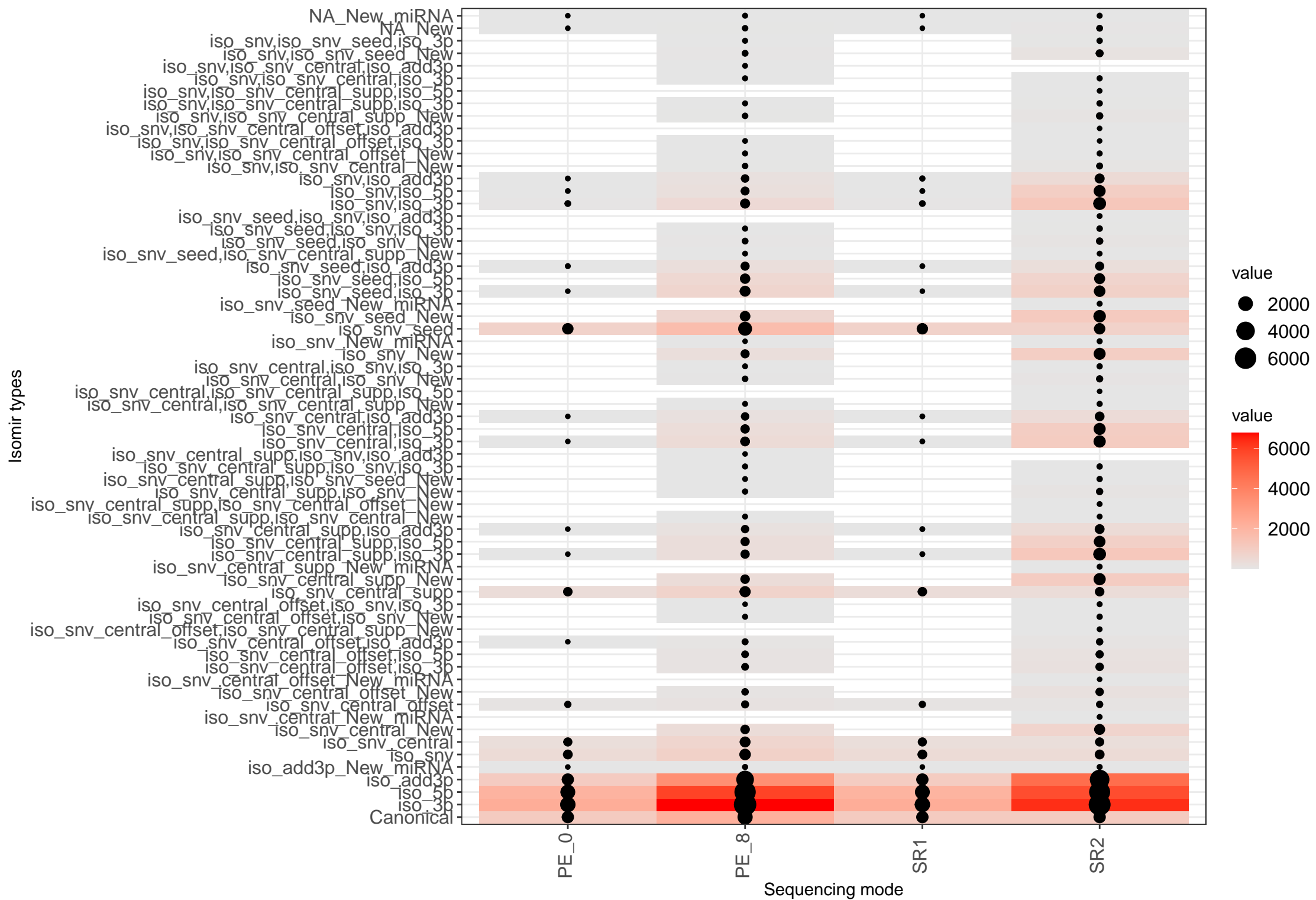

Supplement: Supplementary file 5 — Additional file 5: Figure S3 B: Classification and comparison of miRTOP sequences from simulations results using miraligner software, for each isomiR class and each category of analysis: PE_0 (PE analysis, parameter fastq-join 0% percentage difference); PE_8 (PE analysis, parameter fastq-join 8% percentage difference); SR1 (single end reads R1) and SR2 (SE reads R2). We represented the total average read counts (A) and the count of unique isomiRs detected (B). [file 12859_2021_4128_MOESM5_ESM.pdf]

Average counts per isomir type

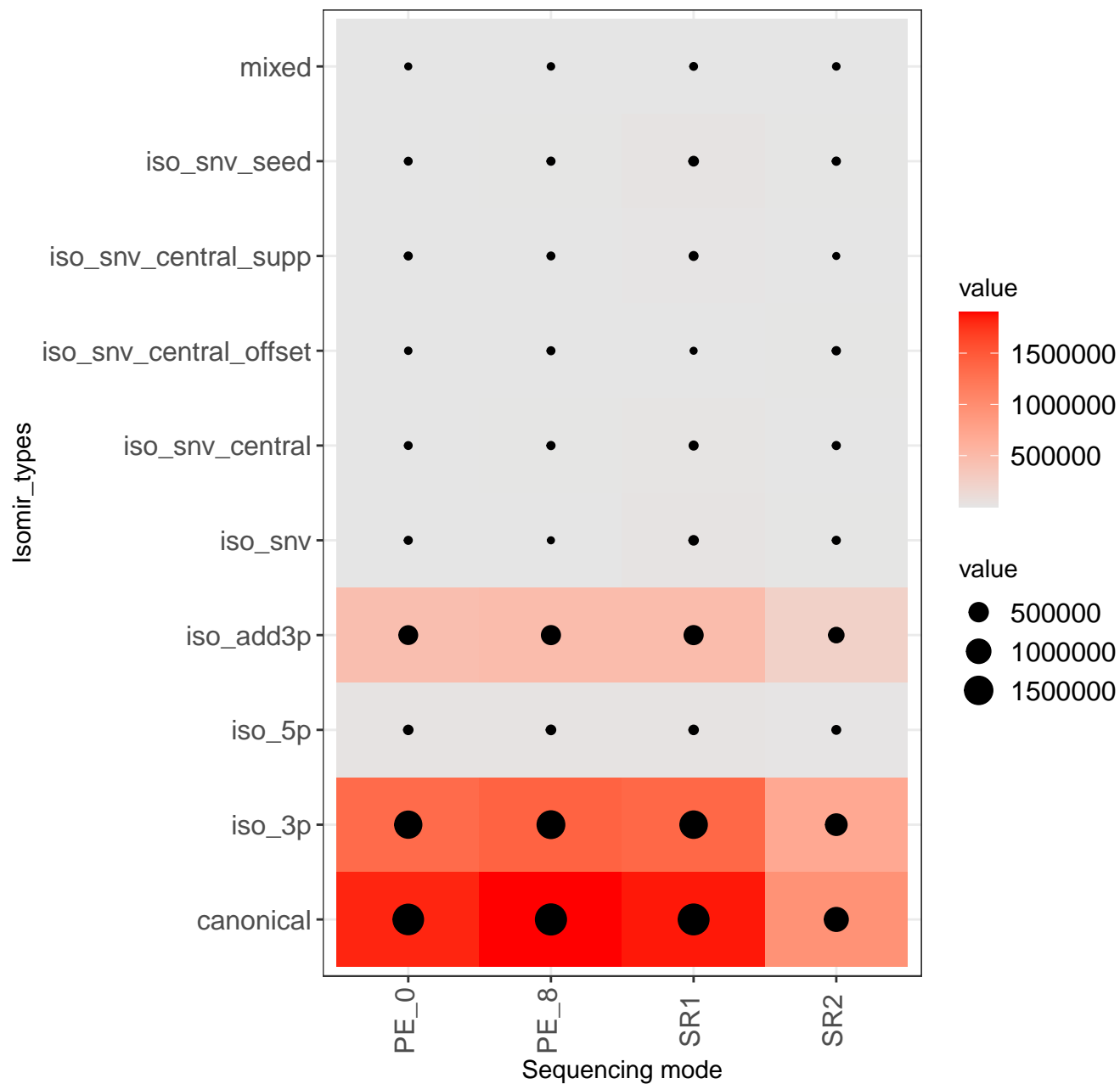

Supplement: Supplementary file 7 — Additional file 7: Figure S5: Overlapping results for each isomiR class category showing of overall read cumulative read counts distribution into isomiR types recognized by each sequencing mode. [file 12859_2021_4128_MOESM7_ESM.pdf]

Average counts per isomir type

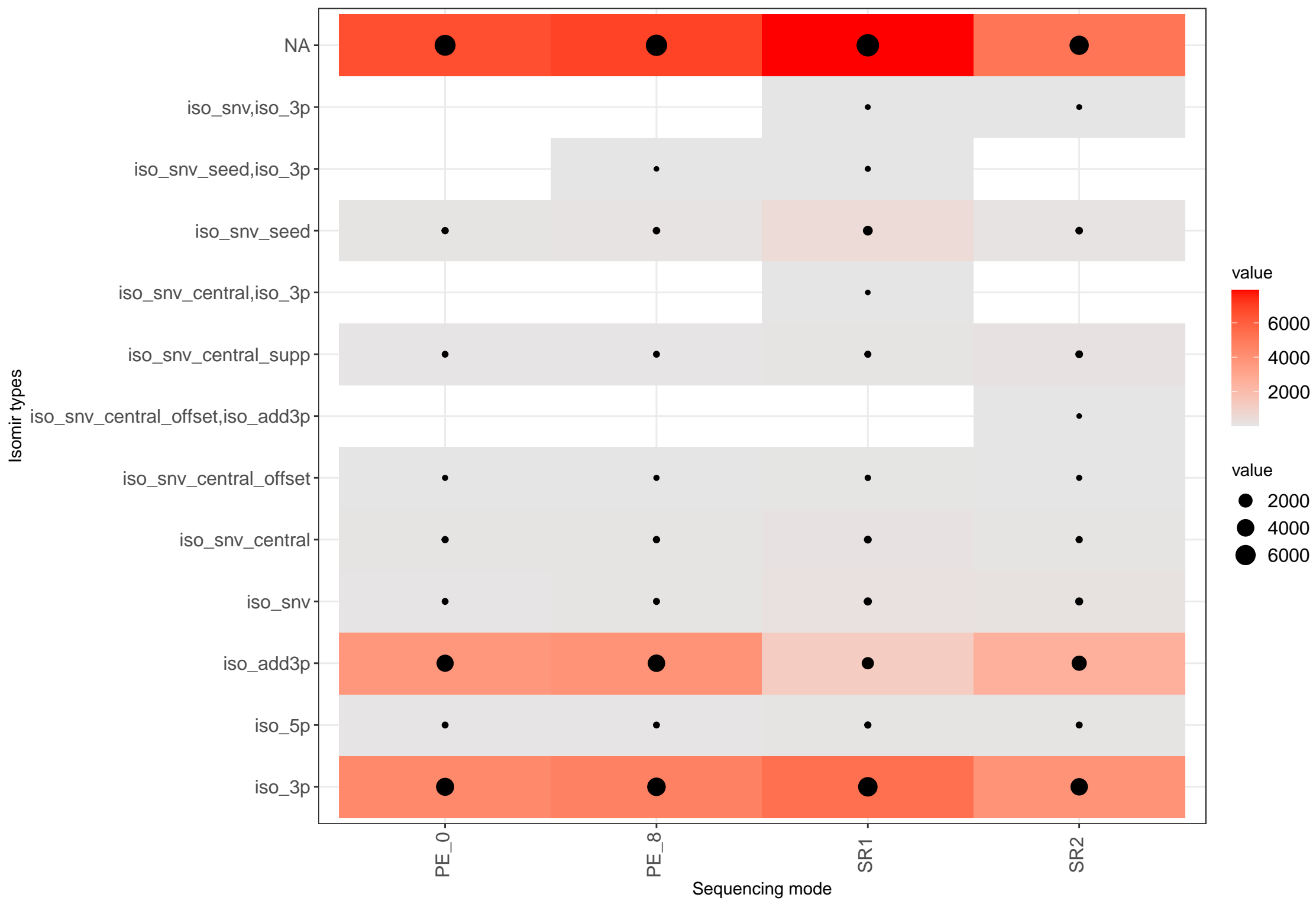

Supplement: Supplementary file 8 — Additional file 8: Figure S6 A: Classification and comparison of miRTOP sequences from GEO project GSE114923 using miraligner software, for each isomiR class and each category of analysis: PE_0 (PE analysis, parameter fastq-join 0% percentage difference); PE_8 (PE analysis, parameter fastq-join 8% percentage difference); SR1 (single end reads R1) and SR2 (SE reads R2). We represented the total average read counts (A) and the count of unique isomiRs detected (B). [file 12859_2021_4128_MOESM8_ESM.pdf]

Unique isomiRs

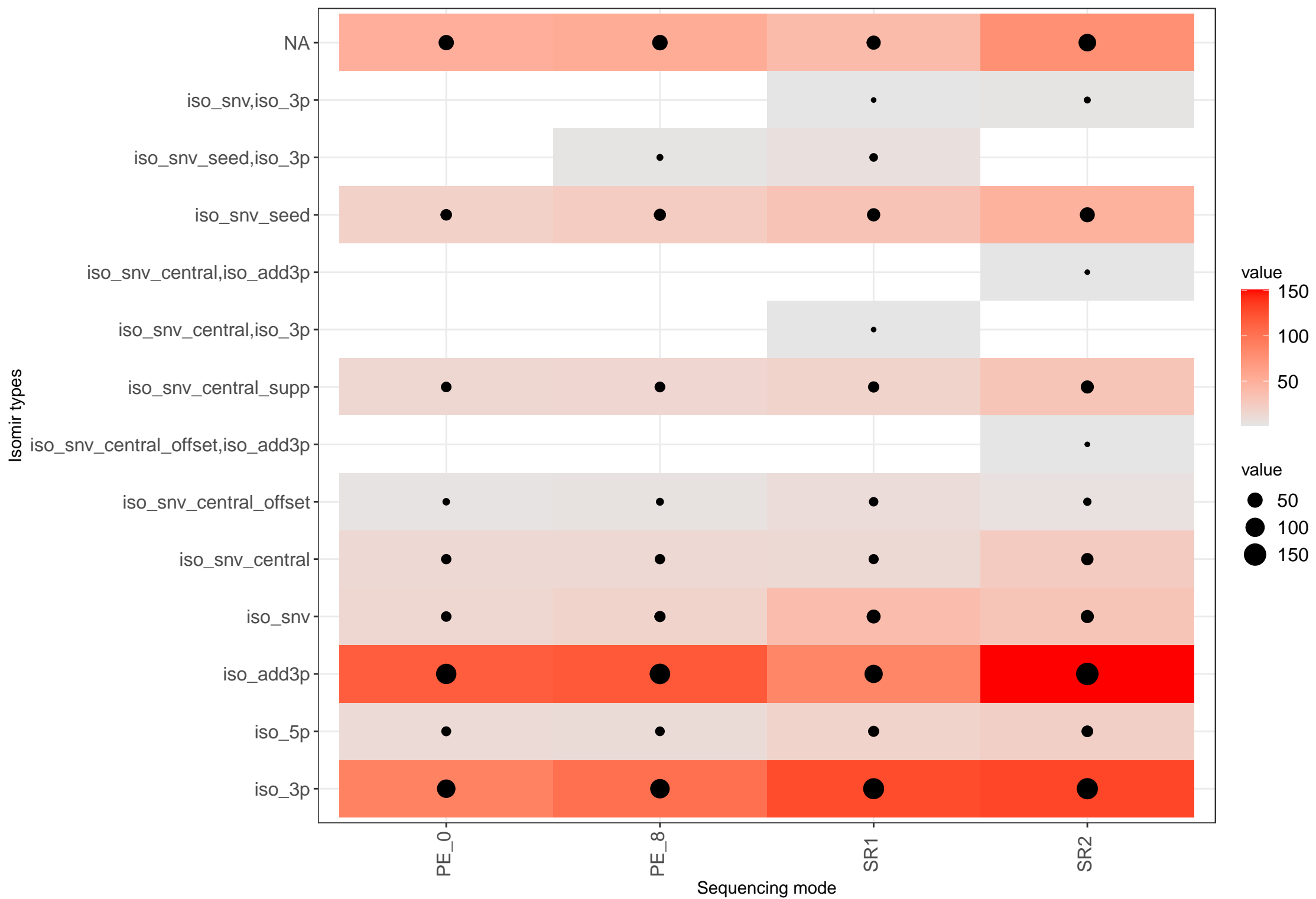

Supplement: Supplementary file 9 — Additional file 9: Figure S6 B: Classification and comparison of miRTOP sequences from GEO project GSE114923 using miraligner software, for each isomiR class and each category of analysis: PE_0 (PE analysis, parameter fastq-join 0% percentage difference); PE_8 (PE analysis, parameter fastq-join 8% percentage difference); SR1 (single end reads R1) and SR2 (SE reads R2). We represented the total average read counts (A) and the count of unique isomiRs detected (B). [file 12859_2021_4128_MOESM9_ESM.pdf]
