# Supplementary material for: Paired-end small RNA sequencing reveals a possible overestimation in the isomiR sequence repertoire previously reported from conventional single read data analysis
Source: BMC Bioinformatics. 2021 Apr 26;22:215. doi: 10.1186/s12859-021-04128-1 (PMC8077951; doi:10.1186/s12859-021-04128-1)

Unique canonical miRTOP isomirs called by method

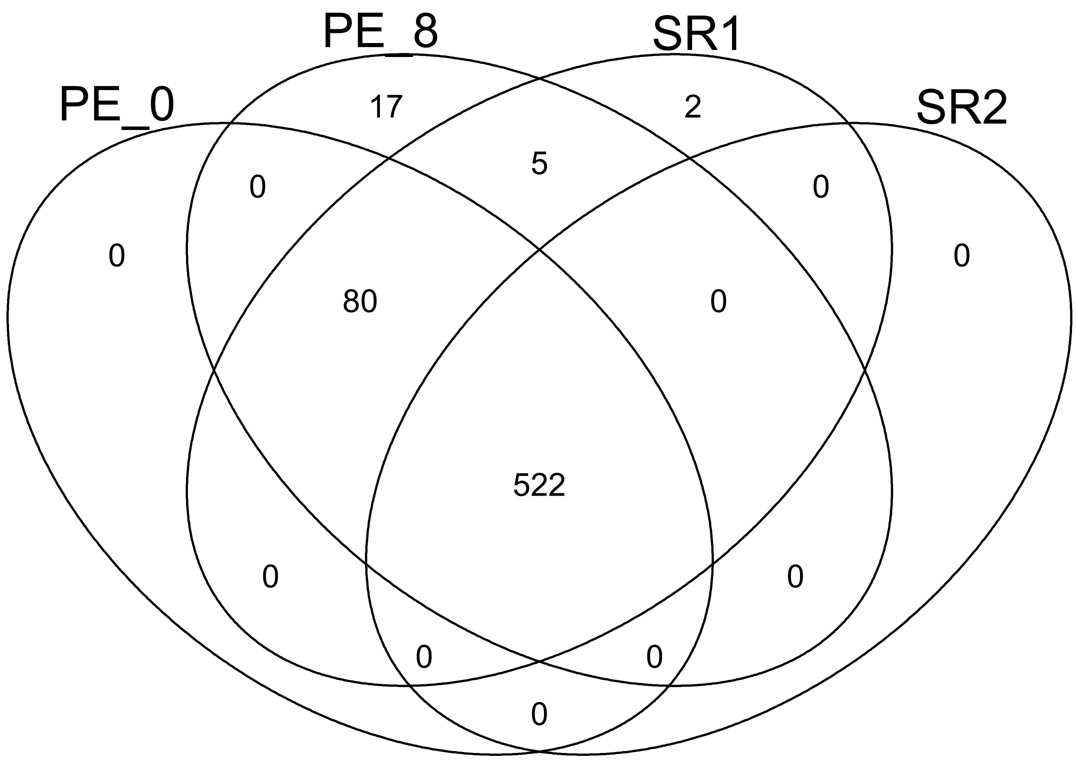

Unique iso_3p miRTOP isomirs called by method

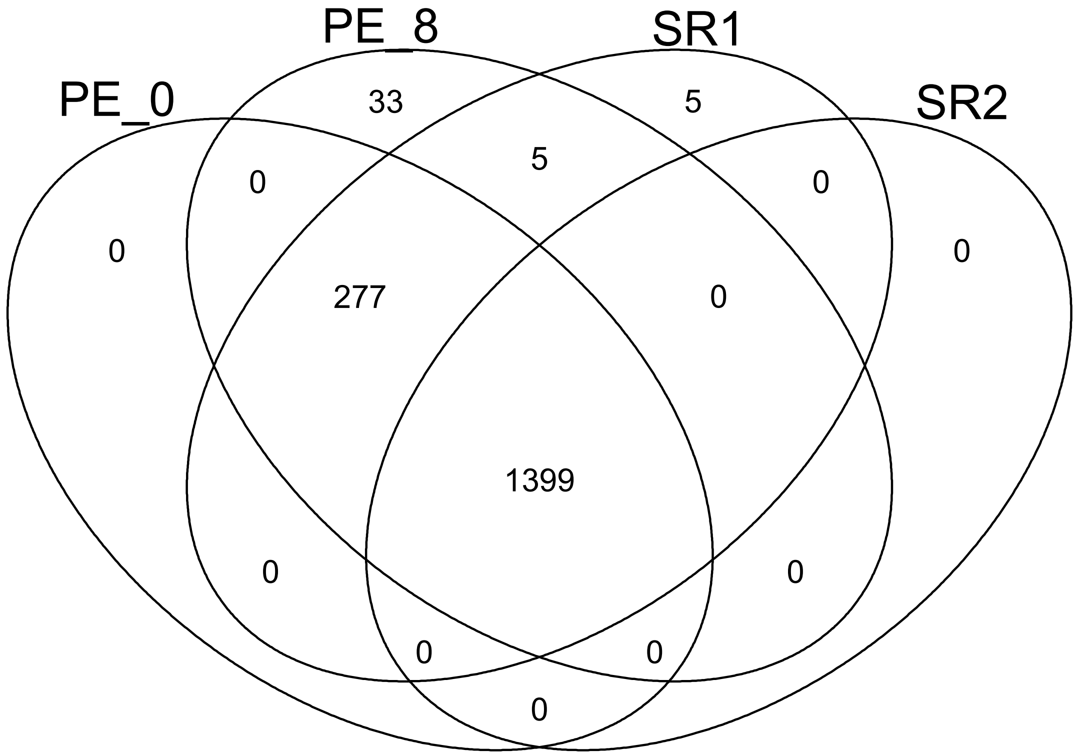

Unique iso_5p miRTOP isomirs called by method

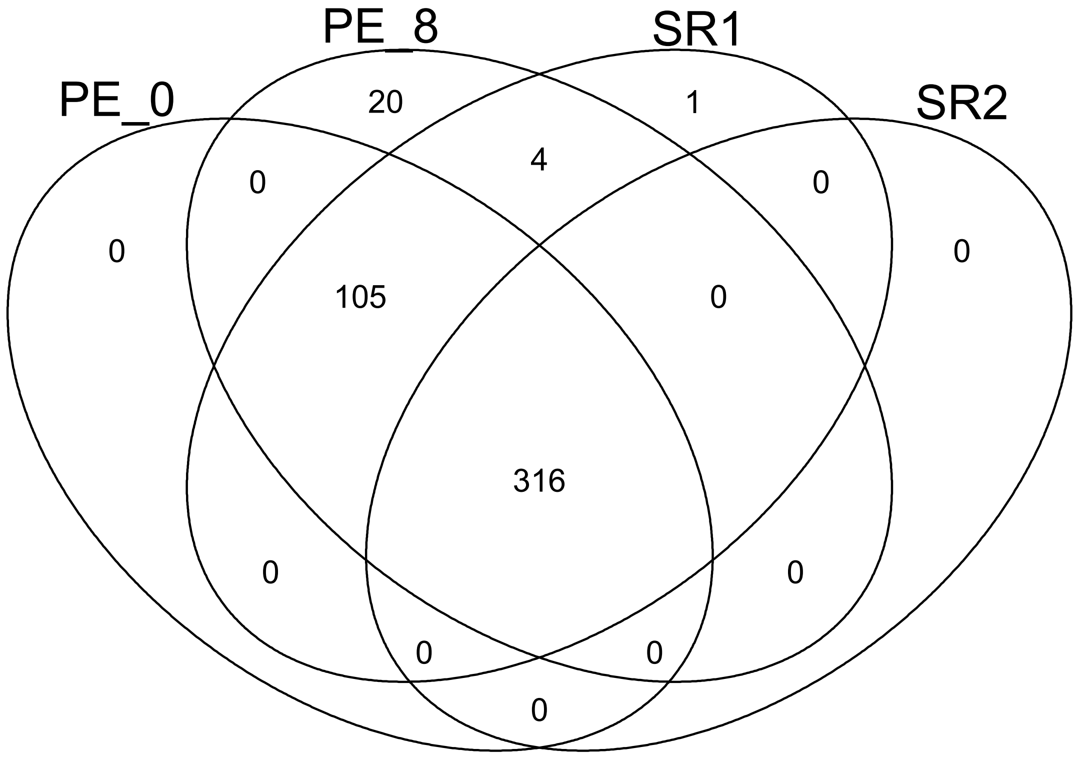

Unique iso_add3p miRTOP isomirs called by method

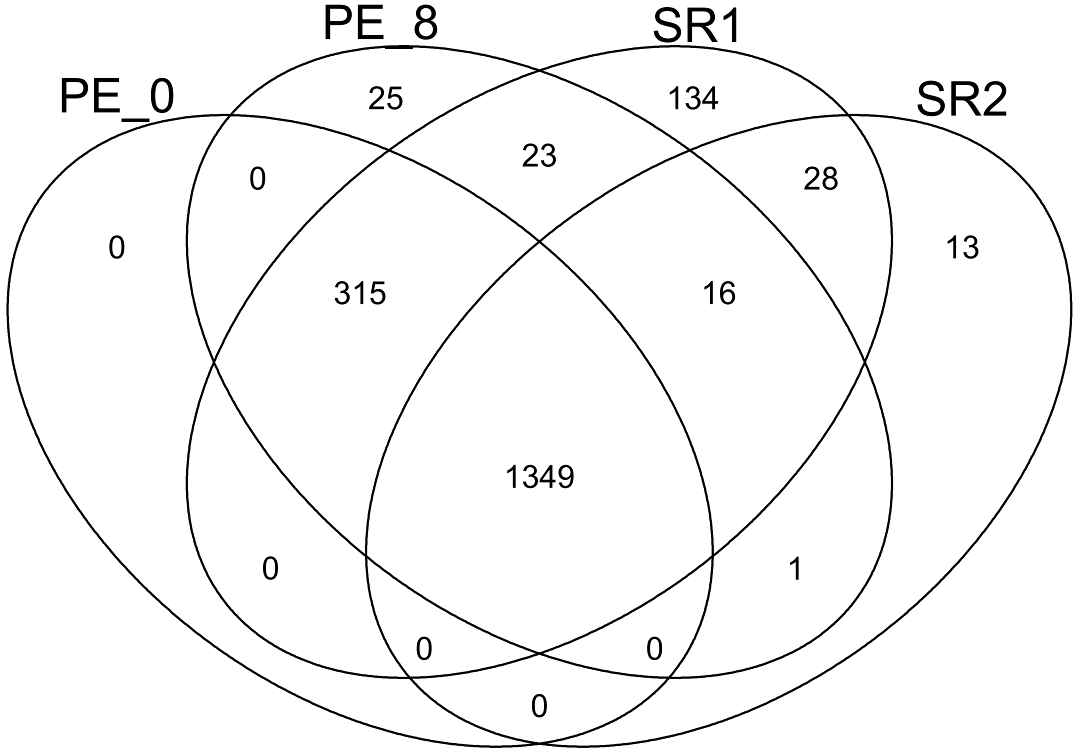

Unique iso_snv miRTOP isomirs called by method

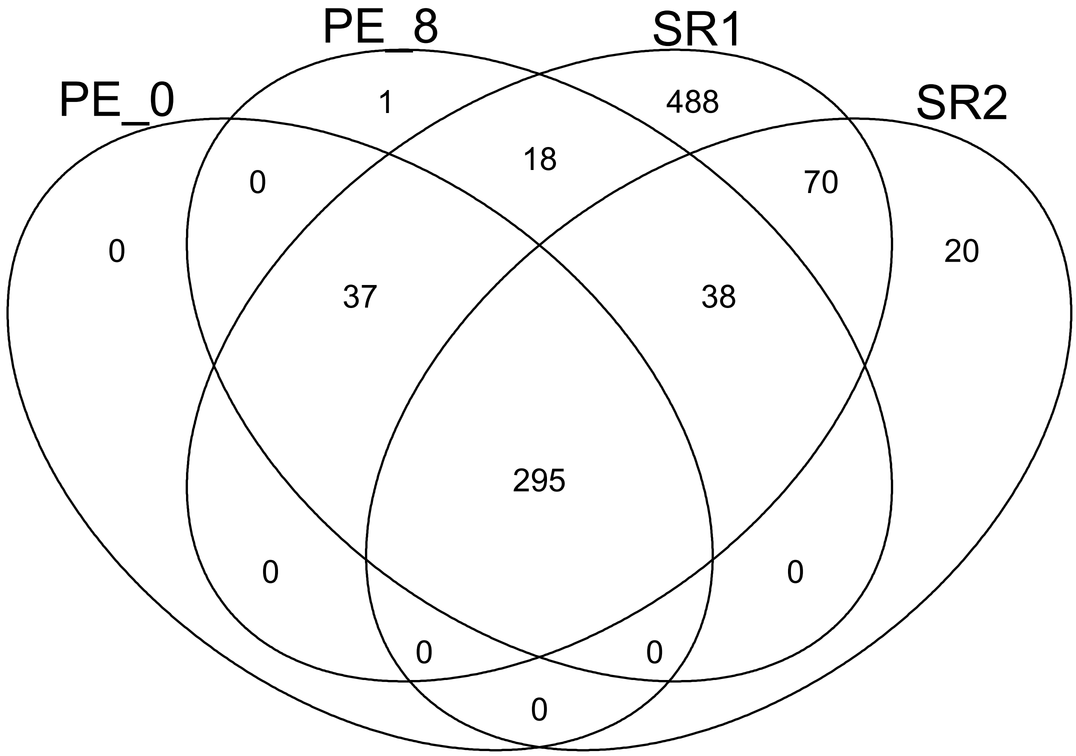

Unique iso_snv_central miRTOP isomirs called by method

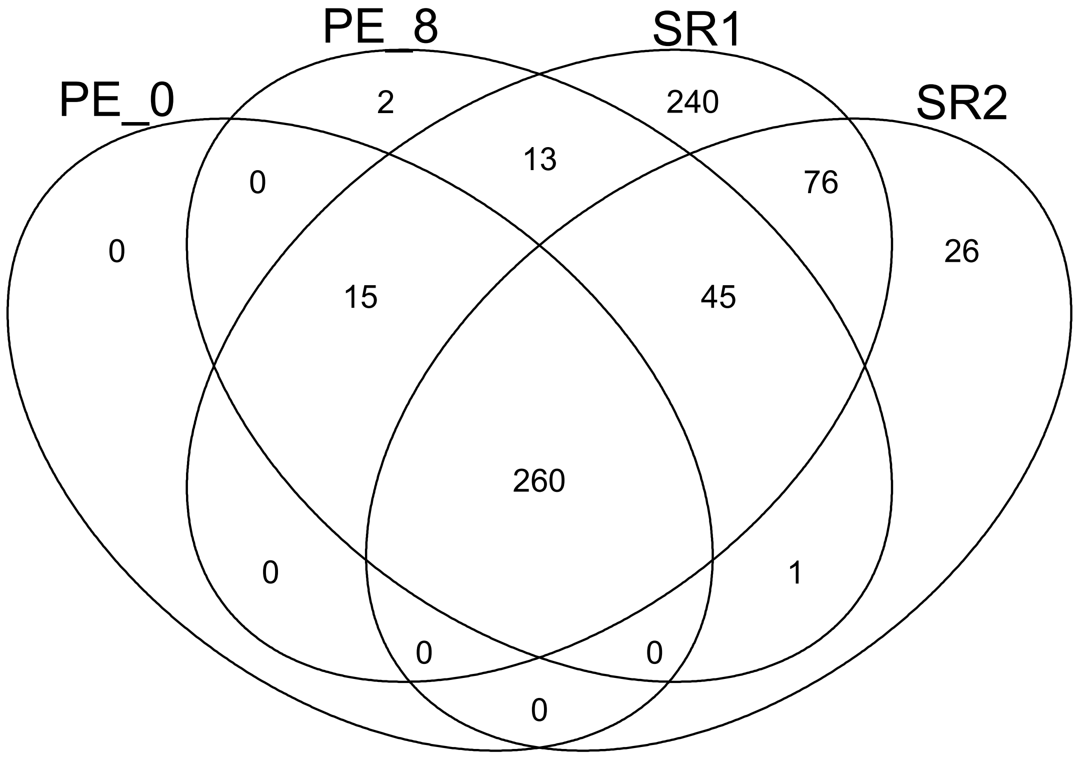

Unique iso_snv_central_offset miRTOP isomirs called by method

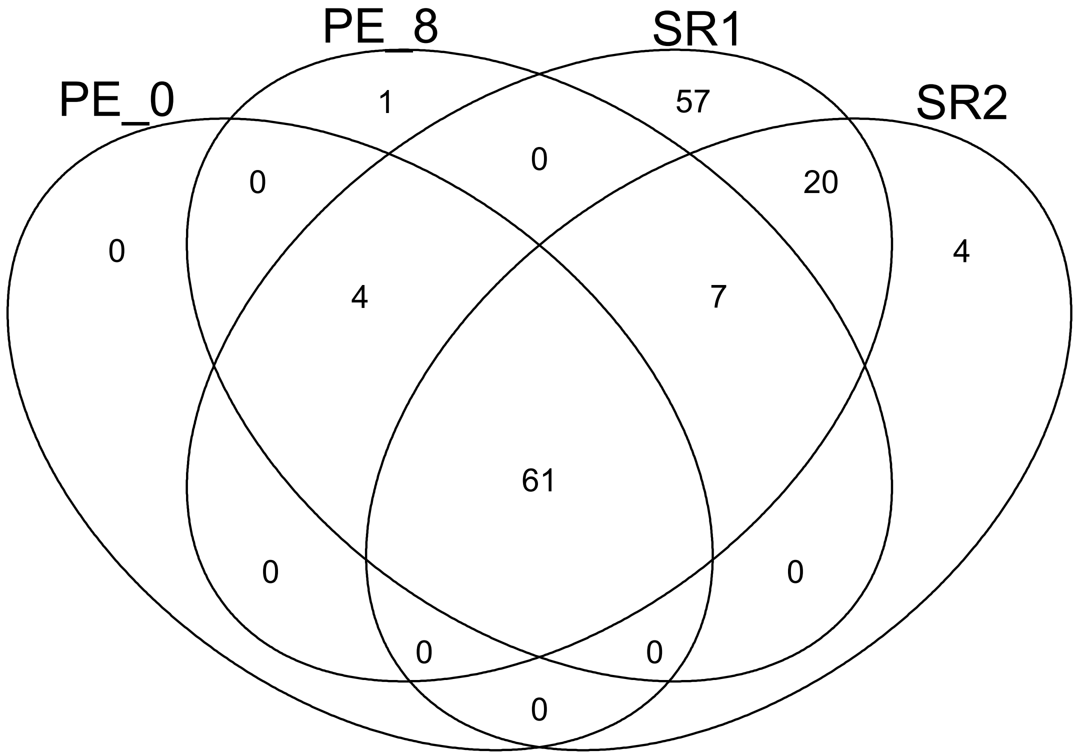

Unique iso_snv_central_supp miRTOP isomirs called by method

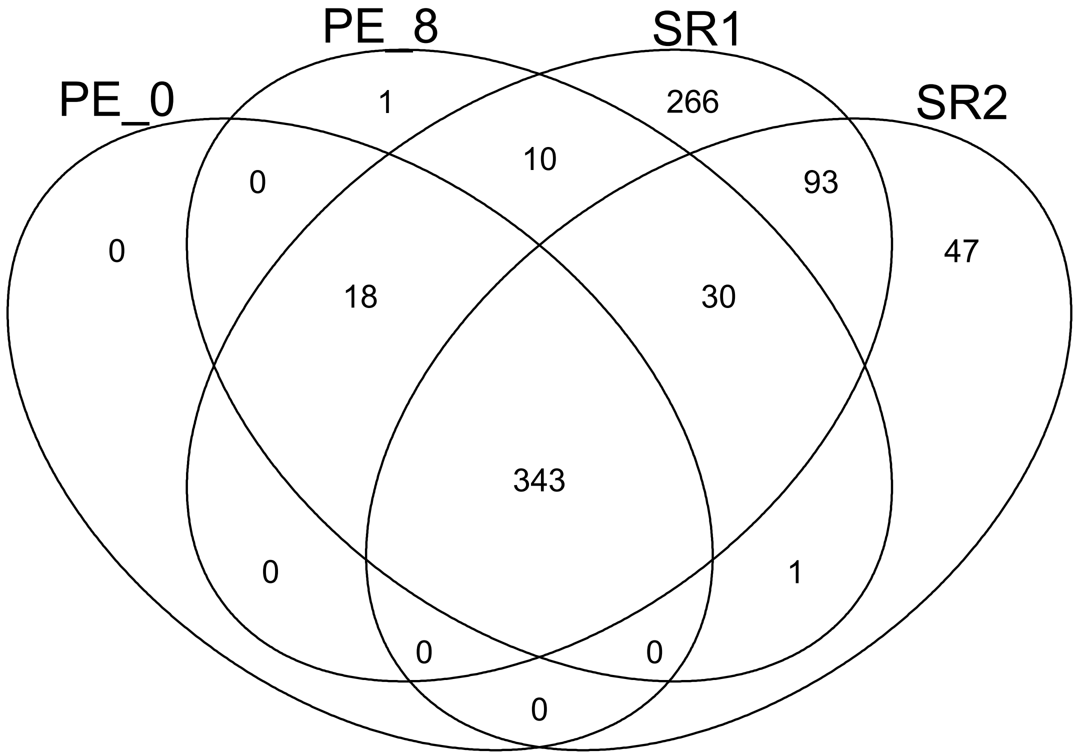

Unique iso_snv_seed miRTOP isomirs called by method

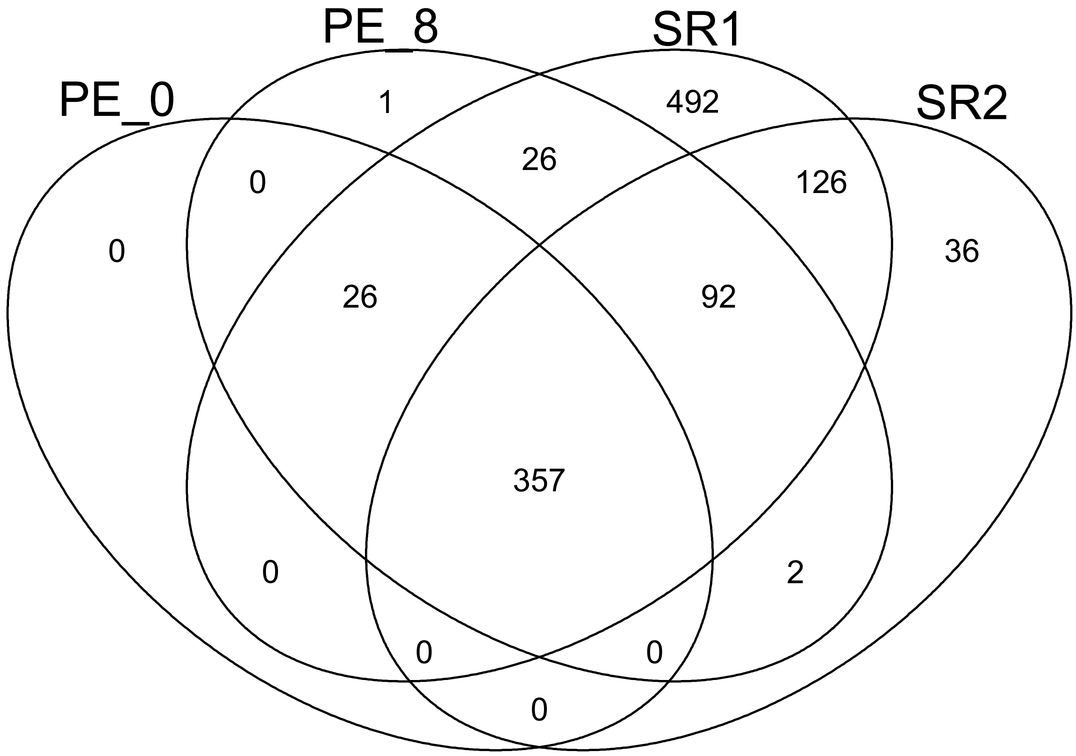

Unique mixed miRTOP isomirs called by method

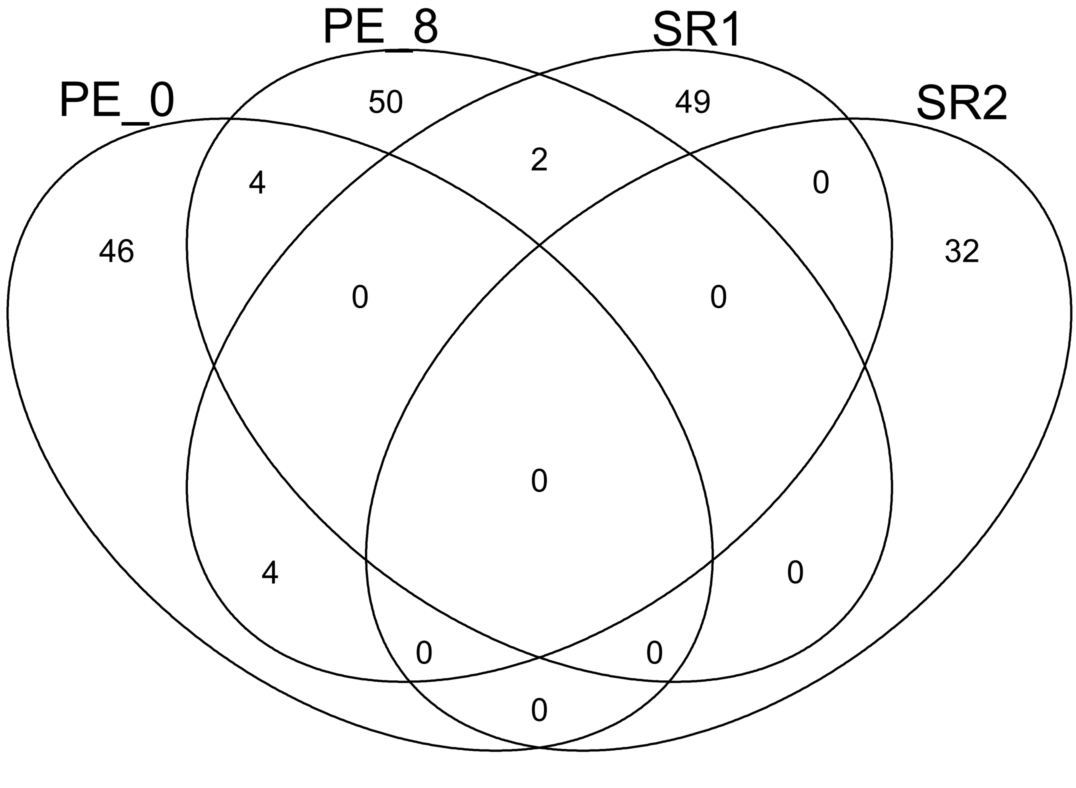

Supplement: Supplementary file 6 — Additional file 6: Figure S4: Venn diagrams of the overlapping isomiRs identified for each category of analysis and subclassified for each isomiR type category: A) canonical; B) iso_3p; C) iso_5p; D) iso_add3p; E) iso_snv; F) iso_snv_central; G) iso_snv_central_offset; H) iso_snv_central_supp; I) iso_snv_central_seed; J) mixed category. [file 12859_2021_4128_MOESM6_ESM.pdf]
