# Supplementary material for: Paired-end small RNA sequencing reveals a possible overestimation in the isomiR sequence repertoire previously reported from conventional single read data analysis
Source: BMC Bioinformatics. 2021 Apr 26;22:215. doi: 10.1186/s12859-021-04128-1 (PMC8077951; doi:10.1186/s12859-021-04128-1)

Supplementary Figure 1

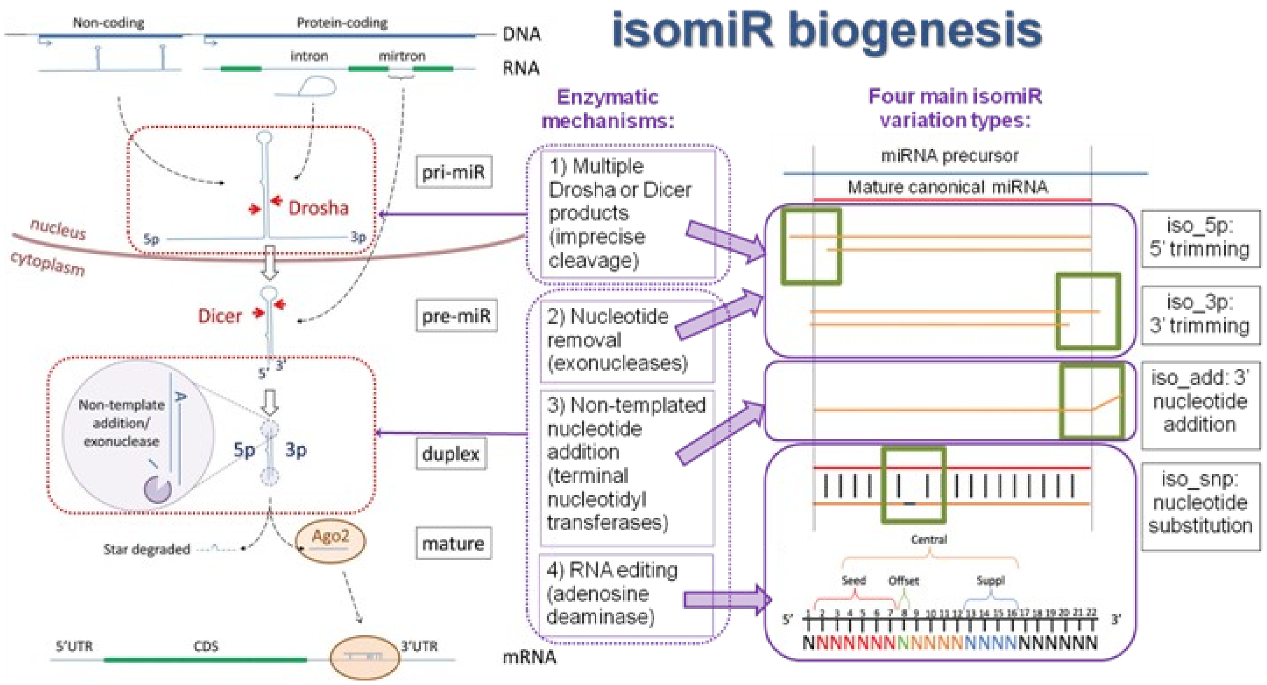

Supplement: Supplementary file 3 [file 12859_2021_4128_MOESM1_ESM.docx]
